# Use of healthcare services at the end of life in decedents compared to their surviving counterparts: A case-control study among adults born before 1946 in Friuli Venezia Giulia

**DOI:** 10.1371/journal.pone.0212086

**Published:** 2019-02-07

**Authors:** Cristina Canova, Paola Anello, Claudio Barbiellini Amidei, Vito Parolin, Loris Zanier, Lorenzo Simonato

**Affiliations:** 1 Department of Cardio-Thoraco-Vascular Sciences and Public Health, University of Padua, Padua, Italy; 2 Epidemiological Service, Health Directorate, Friuli Venezia Giulia Region, Udine, Italy; University Complutense of Madrid, SPAIN

## Abstract

**Background:**

There is a heterogeneous literature on healthcare utilization patterns at the end of life. The objective of this study is to examine the impact of closeness to death on the utilization of acute hospital-based healthcare services and some primary healthcare services and compare differences in gender, age groups and major causes of death disease specific mortality.

**Methods:**

A matched case-control study, nested in a cohort of 411,812 subjects, linked to administrative databases was conducted. All subjects were residents in the Friuli Venezia Giulia Region (Italy), born before 1946, alive in January 2000 and were followed up to December 2014. Overall, 158,571 decedents/cases were matched by gender and year of birth to one control, alive at least one year after their matched case’s death (index-date). Hospital admissions, emergency department visits, drug prescriptions, specialist visits and laboratory tests that occurred 365 days before death/index-date, have been evaluated. Odds Ratios (ORs) for healthcare utilization were estimated through conditional regression models, further adjusted for Charlson Comorbidity Index and stratified by gender, age groups and major causes of death.

**Results:**

Decedents were significantly more likely of having at least one hospital admission (OR 7.0, 6.9–7.1), emergency department visit (OR 5.2, 5.1–5.3), drug prescription (OR 2.8, 2.7–2.9), specialist visit (OR 1.4, 1.4–1.4) and laboratory test (OR 2.7, 2.6–2.7) than their matched surviving counterparts. The ORs were generally lower in the oldest age group (95+) than in the youngest (55–74). Healthcare utilization did not vary by sex, but was higher in subjects who died of cancer.

**Conclusion:**

Closeness to death appeared to be strongly associated with healthcare utilization in adult/elderly subjects. The risk seems to be greater among younger age groups than older ones, especially for acute based services. Reducing acute healthcare at the EOL represents an important issue to improve the quality of life in proximity to death.

## Introduction

Elderly individuals, aged 65 or above, sum up to 15% of the European population [1], but are estimated to contribute to around 30% and 50% of the total healthcare expenditure in Western countries [2]. With a rapidly aging population, a better understanding of healthcare demand in the elderly is becoming crucial to guide healthcare expenditure and policy-making.

Several studies have tried to identify the determinants of healthcare among older adults, but they have arrived to contradictory conclusions. According to some authors, increased healthcare service use, is a pure consequence of ageing [3,4], that is associated to an increase in comorbidities [5, 6].

On the other hand, Zweifel et al. asserted that this conclusion might actually be a”red herring” [7]. According to their analyses, in fact, proximity to death (or end of life period—EOL) is the actual responsible of high healthcare costs and this is phenomenon is greater in older people, simply because they are closer to death than the majority of younger individuals. Despite there being other studies that supported this last interpretation [8,9], a study by Howdon and Rice, suggested that proximity to death is itself a ‘red herring’ that acts as proxy for morbidity [10].

Other researchers found that both proximity to death and age play a role in shaping healthcare costs [11,12].

Overall, there is a certain heterogeneity in literature, on definitions and research objectives concerning healthcare utilization at the EOL. For instance, the timeframe of the EOL itself is not univocal as it ranges from a few months [13–15], to several years before death [16–20]. Furthermore, most studies on healthcare at the EOL, focused on its costs [21–24] and only a limited number considered utilization itself [9,19]. Some studies are concentrated exclusively on decedents that died of specific diseases, such as cancer [25–27] or patients affected by COPD and lung cancer [23], or Alzheimer’s disease [28] and many other conditions.

Throughout these studies, different types of healthcare services have been evaluated. Some studies have investigated only primary healthcare service use [11,15,29], while others focused exclusively on acute healthcare use [17,18,30]. To the best of our knowledge, only few papers considered both acute and long-term care and/or primary care services among decedents and compared it with matching survivors [9,19,31]. According to the abovementioned studies, in the period preceding death, younger-old individuals are more likely to access acute care services compared to older age groups [9,19]. Younger-old decedents compared to survivors, have also shown to have a higher risk of accessing acute care than older decedents [9]. Results concerning primary care use, on the other hand, appear to be contrasting. Nevertheless, none of the identified literature includes acute hospital-based services, along with drug prescriptions and relevant outpatient services, namely laboratory tests and specialist visits, with a matched case-control study design.

The aim of this study is therefore to explore patterns of healthcare utilization (HCU) of both acute hospital-based services (hospitalizations and emergency department visits) and primary healthcare services (drug prescriptions, diagnostic tests and specialist visits), among people aged 55 and above, resident in North Eastern Italy, during their EOL period and to compare it, on an individual level (one-to-one), with that of surviving subjects, matched by age and gender. We will also estimate whether those patterns were or not modified by gender, class ages and major causes of death

## Methods

### Setting and study population

Italy has a tax-based National Health Service (NHS) system, which provides universal coverage, like in most European countries. The NHS delivers healthcare free or upon co-payment to all Italian and European Union citizens. This is granted to all subjects who have the right to access NHS care, regardless of income. Healthcare in the Italian NHS, is significantly decentralized to the Regions, which have a vast autonomy in the administration and organization of healthcare in their own territory. Data concerning healthcare utilization is recorded at a Regional level, for all residents assisted by the NHS, even when patients access healthcare services outside the Region where they reside.

This study was conducted in the Region of Friuli Venezia Giulia, Italy, with a total population of about 1.2 million. This Region has an automated centralized system developed in the ‘80s with the objective of systematically collecting and pooling data on healthcare funded by the National Health Service using a unique anonymous ID regional code.

### Definition of the cohort

A matched case-control design, nested in a cohort, linked to administrative data, was adopted. The original cohort included 411,812 subjects born before 1946 and alive in January 2000, resident in the Friuli Venezia Giulia Region, followed-up from January 2001 to December 2014. Data on the population derives from the **Health Population register** that contains demographic information on all residents in the Region, who have the right to be assisted by the NHS.

All the subjects who died in the follow-up period (cases-decedents) were matched by gender and year of birth to one control (survivor) who was alive at least one year after the counterpart’s death. The purpose was to ensure same age and gender distribution in decedents and survivors.

By adopting a SAS macro [32] through an iterative process, all cases were identified from the cohort. In the same population, for each case, all possible controls were identified, according to the following criteria: having the same gender, year of birth and being alive at least 12 months after the matching case’s death. Among the controls, there could be subjects identified as cases in the following years (the same way as cases could have been eligible to be controls for a previous case). One control for each case was randomly selected. This way, each control was assigned a date to end the follow-up (index date), that corresponded to the date of death of the matched case.

At the end of the iterative loop, 158,611 cases have been identified and 158,571 of them had a matching counterpart. Only 40 decedents did not have a matched control and were eliminated from the study.

### Healthcare use

The databases used, concerned both acute hospital-based services and primary care services. Acute hospital-based service databases consisted of: **hospital admissions (HA),** which hold information collected during any episode of inpatient care, within or outside the Region, with up to six diagnostic codes (according to the International Classification of Diseases Ninth Revision—ICD-IX), recorded at discharge; **emergency department (ED)** visits, which hold data on patients that accessed an emergency department, with the specific triage color tag (from green to black color tags, with the addition of white tags for subjects that would have not require an ED visit). Primary care services consisted of databases on: **drug prescriptions (DP)** where all dispensations of NHS-reimbursable drugs are registered (coded by the Anatomical Therapeutic Chemical -ATC- code); **ambulatory care service database** which holds outpatient visits for diagnostic, therapeutic or rehabilitation services, that was further divided in **specialist visits (SV)** and **laboratory tests (LT)**. These Regional registries were linked to the cases and controls using the anonymized ID number, in order to detect all the events that occurred 365 days before the index date (case’s date of death).

All HAs that presented “death” as discharge modality, have been eliminated. All ED visits with black triage codes (patient dead at admission to the ED) and all ED visits that subsequently led to a hospitalization where the patient died have also been removed.

### Statistical analyses

The first analyses evaluated the proportion of cases and controls that used anyone of the five types of healthcare services at least once (any vs none). Secondly, the use of each healthcare service was considered as categorical variable (quartiles of utilization) defining specific cut-off through the quartiles of utilization among controls

The analyses were performed on the entire study population and stratified by gender, age groups (55–74, 75–84, 85–94, 95+ years old) and major causes of death (neoplasms, circulatory disease, respiratory diseases) of cases. The age among cases was calculated at the date of death. The corresponding age among controls was calculated at the as the index_date, namely the date when the matching control died. When stratifying by age, 4678 paired couples of cases and controls—9356 subjects–(2.95% of the sampled population) have been eliminated, because they belonged to different age classes, since they were matched by year of birth, but the age was calculated at the date of death/index date.

Conditional binomial regression models were used to estimate Odds Ratios (ORs) with 95% confidence intervals (CI) for the use of each healthcare service (as dichotomous and categorical variables), among the one-to-one matched case-control pairs. Analyses were further adjusted by Charlson Comorbidity Index [33]. The Charlson Comorbidity Index was calculated by using the ICD-IX coded primary diagnosis of hospital discharge records, from the 12 months before date of death/index date. The index is calculated by summing the weights (1 to 6) assigned to a list of 17 medical conditions that have led to a hospital admission. The so obtained index has been divided in four categories: 0, 1 or 2, 3 or 4, and 5 or more.

## Results

Overall, 317,142 individuals, composed of 158,571 decedents and an equal amount of matched controls were included in the study. The distribution by gender, age, comorbidity index and cause of death is shown in Table 1. Out of all the pairs, 44.70% were men and 55.30% women. The majority of subjects belonged to the age groups 75–84 (34.23%) and 85–94 (37.05%). The Charlson Comorbidity Index varied among cases and controls, with decedents having more comorbidities than survivors. The major causes of death were related to diseases of the circulatory system (39.31%), followed by neoplasms (29.15%) and diseases of the respiratory system (9.40%).

**Table 1:** Characteristics of the cases (decedents) and matched controls (survivors).

Variable	Cases (n = 158,571)	Control (n = 158,571)
Gender		
Male	70877 (44.70)	70877 (44.70)
Female	87694 (55.30)	87694 (55.30)
Age at the “Index_date”		
55–74	32533 (20.51)	32620 (20.57)
75–84	54282 (34.23)	54371 (34.29)
85–94	58743 (37.05)	58618 (36.97)
95+	13013 (8.21)	12962 (8.17)
Comorbidity Index		
0	95797 (60.41)	147733 (93.16)
1–2	50055 (31.57)	10246 (6.46)
3–4	6186 (3.90)	472 (0.30)
≥5	6533 (4.12)	120 (0.08)
Cause of Death ICD IX code		
Infectious and Parasitic Diseases 001–139	2180 (1.37)	//
Neoplasms 140–239	46219 (29.15)	
Endocrine, Nutritional and Metabolic Diseases 240–279	3816 (2.41)	
Diseases of the Blood 280–289	607 (0.38)	
Mental Disorders 290–319	4258 (2.69)	
Diseases of the Nervous System 320–389	3769 (2.38)	
Diseases of the Circulatory System 390–459	62341 (39.31)	
Diseases of the Respiratory System 460–519	14906 (9.40)	
Diseases of the Digestive System 520–579	7406 (4.67)	
Diseases of the Genitourinary System 580–629	2483 (1.57)	
Diseases of the Skin 680–709	211 (0.13)	
Diseases of the Musculoskeletal System 710–739	1168 (0.74)	
Symptoms, Signs, and Ill-defined Conditions 780–799	4758 (3.00)	
Injury 800–999	3831 (2.42)	
Without Causes	613 (0.39)	

Healthcare utilization during the one-year study period, among cases and controls, stratified by gender, is shown in Table 2.

**Table 2:** Healthcare utilization during the last year of life by gender; Odds Ratios (OR) and 95% Confidence Intervals (CI) from conditional logistic regression for cases (decedents) and matched controls (survivors).

	Male (n = 141754)				Female (n = 175388)				Total (n = 317142)			
	Cases	Controls			Cases	Controls			Cases	Controls		
	%	%	OR (95% CI)	adjOR (95% CI)	%	%	OR (95% CI)	adjOR (95% CI)	%	%	OR (95% ICI)	adjOR (95% CI)
Hospitalization admission												
At least one HA	66.23	20.43	7.5 (7.3–7.7)	4.0 (3.9–4.2)	58.92	17.75	6.6 (6.4–6.8)	4.0 (3.9–4.1)	62.19	18.95	7.0 (6.9–7.1)	4.0 (3.9–4.1)
Total HA by classes												
0	33.77	79.57	1	1	41.08	82.25	1	1	37.81	81.05	1	1
1	26.80	14.13	4.4 (4.2–4.5)	3.2 (3.1–3.3)	28.56	12.92	4.4 (4.3–4.6)	3.3 (3.2–3.4)	27.77	13.46	4.4 (4.3–4.5)	3.3 (3.2–3.4)
2	17.50	4.22	9.7 (9.2–10.2)	5.8 (5.4–6.1)	15.48	3.43	8.9 (8.5–9.4)	5.5 (5.2–5.8)	16.38	3.78	9.3 (9.0–9.6)	5.6 (5.4–5.9)
3+	21.94	2.09	24.0 (22.5–25.6)	11.4 (10.6–12.3)	14.87	1.40	20.6 (19.3–22.0)	10.1 (9.4–10.9)	18.03	1.71	22.3 (21.3–23.4)	10.8 (10.3–11.4)
Emergency department visits												
At least one ED	65.07	25.40	5.68 (5.5–5.8)	3.5 (3.4–3.6)	61.79	25.46	4.9 (4.8–5.0)	3.2 (3.1–3.2)	63.26	25.43	5.2 (5.1–5.3)	3.3 (3.2–3.4)
Total ED by classes												
0	34.93	74.60	1	1	38.21	74.54	1	1	36.74	74.57	1	1
1	26.07	16.50	3.5 (3.4–3.6)	2.6 (2.5–2.7)	27.27	16.62	3.3 (3.2–3.4)	2.5 (2.4–2.6)	26.73	16.57	3.3 (3.3–3.4)	2.5 (2.5–2.6)
2+	38.99	8.91	9.8 (9.5–10.2)	5.2 (5.0–5.4)	34.53	8.83	8.0 (7.7–8.2)	4.5 (4.3–4.7)	36.52	8.87	8.7 (8.5–9.0)	4.8 (4.7–4.9)
Drug prescriptions												
At least one DP	95.59	86.33	3.5 (3.3–3.7)	2.4 (2.3–2.4)	93.30	85.37	2.4 (2.4–2.5)	1.9 (1.8–2.0)	94.32	85.80	2.8 (2.7–2.9)	2.1 (2.0–2.1)
Total DP by classes												
0–4	10.63	24.50	1	1	15.59	25.57	1	1	13.37	25.09	1	1
5–15	16.10	22.66	1.7 (1.6–1.8)	1.4 (1.7–1.8)	19.26	24.67	1.3 (1.3–1.3)	1.2 (1.1–1.2)	17.85	23.78	1.4 (1.4–1.5)	1.3 (1.2–1.3)
16–31	23.41	24.05	2.4 (2.3–2.5)	1.4 (1.4–2.8)	24.79	25.25	1.6 (1.6–1.7)	1.3 (1.3–1.4)	24.17	24.72	1.9 (1.9–1.9)	1.5 (1.5–1.5)
32+	49.86	28.79	4.3 (4.2–4.5)	2.7 (2.6–2.8)	40.36	24.50	2.8 (2.8–2.9)	2.0 (1.9–2.0)	44.60	26.42	3.4 (3.3–3.5)	2.2 (2.2–1.3)
Specialist visits												
At least one SV	63.64	53.27	1.6 (1.5–1.6)	1.2 (1.1–1.2)	47.05	41.63	1.3 (1.3–1.3)	1.0 (1.0–1.0)	54.46	46.84	1.4 (1.4–1.4)	1.1 (1.1–1.1)
Total SV by classes												
0	36.36	46.73	1	1	52.95	58.37	1	1	45.54	53.16	1	1
1	17.48	18.64	1.2 (1.2–1.3)	1.1 (1.0–1.1)	16.91	17.09	1.1 (1.1–1.1)	1.0 (1.0–1.0)	17.16	17.78	1.2 (1.1–1.2)	1.0 (1.0–1.0)
2	10.83	11.24	1.3 (1.2–1.3)	1.0 (1.0–1.1)	8.57	8.37	1.2 (1.1–1.2)	1.0 (0.9–1.0)	9.58	9.66	1.2 (1.2–1.2)	1.0 (1.0–1.0)
3+	35.32	23.40	2.0 (2.0–2.1)	1.3 (1.3–1.4)	21.57	16.17	1.5 (1.5–1.6)	1.1 (1.0–1.1)	27.72	19.40	1.7 (1.7–1.8)	1.2 (1.2–1.2)
Laboratory tests												
At least one LT	83.10	65.92	2.6 (2.6–2.7)	1.9 (1.9–2.0)	78.09	58.16	2.7 (2.6–2.7)	2.1 (2.1–2.2)	80.33	61.63	2.7 (2.6–2.7)	2.0 (2.0–2.1)
Total LT by classes												
0	16.90	34.08	1	1	21.91	41.84	1	1	19.67	38.37	1	1
1–2	16.89	22.44	1.6 (1.5–1.6)	1.4 (1.3–1.4)	21.15	23.71	1.8 (1.7–1.8)	1.6 (1.6–1.7)	19.25	23.14	1.7 (1.6–1.7)	1.5 (1.5–1.5)
3–4	15.82	18.40	1.8 (1.8–1.9)	1.5 (1.5–1.6)	16.85	16.30	2.1 (2.0–2.2)	1.8 (1.8–1.9)	16.39	17.24	2.0 (2.0–2.0)	1.7 (1.7–1.7)
5+	50.39	25.08	4.4 (4.3–4.5)	2.8 (2.7–2.9)	40.08	18.14	4.7 (4.5–4.8)	3.2 (3.1–3.3)	44.69	21.24	4.6 (4.5–4.7)	3.1 (3.0–3.1)

adj: adjustment for Charlson Comorbidity Index.

Healthcare utilization among decedents compared to survivors was higher with a statistical significance, especially regarding hospital admissions (OR = 7; 95% CI 6.9–7.1) and emergency department visits (OR = 5.2; 95% CI 5.1–5.3), while in a less extent for drug prescriptions (OR = 2.8; 95% CI 2.7–2.9), laboratory tests (OR = 2.7; 95% CI 2.6–2.7) and specialist visits (OR = 1.4; 95% CI 1.4–1.4). Decedents were significantly more likely than their matching controls, to have a higher number of each service utilization, across all considered quartiles of utilization (p-trend <0.001).

Odds Ratios adjusted for Charlson Comorbidity Index decreased in value, while keeping the same trends and statistical significance as the non-adjusted ones, except for specialist visits (OR = 1.1; 95% CI. 1.1–1.1).

Patterns of utilization were relatively similar among male and female individuals, although the risks of HCU among the female decedents compared to their matched controls were always lower than males, especially for drug prescriptions, with the exception of laboratory tests.

Table 3 describes HCU stratified by age groups. The rate of decedents who were hospitalized at least once, decreased with age, reaching the lowest value in the very elderly (from 70% among the 55–75 age group to 45% among the 95+), with more stays and days per stay during hospitalizations among younger decedents (21.9 days among 55–75 vs 7.9 days among 95+, data not shown in the tables). Similar patterns were seen for specialist visits, with a marked reduction in the utilization, with the increase of age (from 72% to 25%).

**Table 3:** Healthcare utilization during the last year of life by age groups; Odds Ratios (OR) and 95% Confidence Intervals (CI) from conditional logistic regression for cases (decedents) and matched controls (survivors).

	Age 55–74 (n = 63764)				Age 75–84 (n = 105118)				Age 85–94 (n = 114072)				Age 95+ (n = 24832)			
	Cases	Controls			Cases	Controls			Cases	Controls			Cases	Controls		
	%	%	OR (95% CI)	adjOR(95% IC)	%	%	OR (95% CI)	adjOR(95% CI)	%	%	OR (95% CI)	adjOR(95% CI)	%	%	OR (95% CI)	adjOR(95% CI)
Hospitalization admission			** **	** **			** **	** **			** **	** **			** **	** **
At least one HA	69.90	15.12	12.8 (12.1–13.4)	4.8 (4.5–5.1)	66.16	19.90	7.4 (7.7–8.2)	4.2 (4.0–4.3)	58.05	20.90	5.2 (5.0–5.4)	3.6 (3.5–3.8)	44.97	15.18	4.7 (4.4–5.1)	3.9 (3.6–4.2)
Total HA by classes																
0	30.10	84.88	1	1	33.84	80.10	1	1	41.95	79.10	1	1	55.03	84.82	1	1
1	23.53	10.76	6.0 (5.7–6.4)	3.6 (3.4–3.8)	27.94	13.69	4.9 (4.7–5.1)	3.4 (3.2–3.5)	30.24	15.12	3.7 (3.6–3.9)	3.1 (3.0–3.2)	27.05	11.36	3.8 (3.5–4.1)	3.5 (3.2–3.8)
2	17.73	2.96	16.8 (15.3–18.4)	7.0 (6.3–7.8)	17.53	4.19	10.0 (9.4–10.6)	5.5 (5.2–5.9)	15.61	4.05	7.2 (6.8–7.6)	5.2 (4.9–5.5)	11.27	2.79	6.5 (5.7–7.4)	5.5 (4.7–6.3)
3+	28.64	1.41	55.6 (49.4–62.6)	16.6 (14.5–18.9)	20.69	2.02	24.3 (22.5–26.2)	10.8 (9.9–11.8)	12.20	1.72	13.3 (12.4–14.4)	8.3 (7.7–9.1)	6.65	1.03	10.3 (8.4–12.5)	7.9 (6.3–9.8)
Emergency department visits																
At least one ED	61.65	19.24	7.1 (6.8–7.4)	3.7 (3.5–3.9)	65.79	25.67	5.8 (5.6–6.0)	3.5 (3.3–3.6)	63.46	29.10	4.3 (4.2–4.5)	3.1 (3.0–3.2)	55.63	22.91	4.3 (4.1–4.6)	3.5 (3.3–3.8)
Total ED by classes																
0	38.35	80.76	1	1	34.21	74.33	1	1	36.54	70.90	1	1	44.37	77.09	1	1
1	25.72	13.51	4.2 (4.0–4.4)	2.8 (2.6–2.9)	26.55	16.83	3.5 (3.4–3.7)	2.6 (2.5–2.7)	27.32	18.38	2.9 (2.8–3.0)	2.4 (2.3–2.5)	27.35	14.55	3.4 (3.1–3.6)	3.0 (2.8–3.2)
2+	35.93	5.72	14.0 (13.1–15.0)	5.9 (5.5–6.3)	39.24	8.85	10.2 (9.8–10.7)	5.3 (5.1–5.6)	36.14	10.72	6.7 (6.5–7.0)	4.4 (4.2–4.6)	28.28	8.35	6.0 (5.5–6.6)	4.6 (4.8–5.0)
Drug prescriptions																
At least one DP	94.56	84.11	3.3 (3.1–3.5)	1.9 (1.7–2.0)	95.84	90.72	2.4 (2.2–2.5)	1.6 (1.5–1.7)	93.98	86.89	2.4 (2.3–2.5)	1.9 (1.8–2.0)	88.85	63.84	4.6 (4.3–5.0)	4.1 (3.8–4.4)
Total DP by classes																
0–4	12.28	32.38	1	1	10.24	18.73	1	1	14.58	22.50	1	1	23.88	46.13	1	1
5–15	18.00	28.32	1.8 (1.7–1.9)	1.3 (1.2–1.4)	16.21	24.20	1.2 (1.2–1.3)	1.0 (1.0–2.1)	18.21	22.01	1.3 (1.2–1.3)	1.2 (1.1–1.2)	22.72	18.63	2.3 (2.2–2.6)	2.2 (2.1–2.4)
16–31	24.35	22.30	3.1 (2.9–3.3)	2.0 (1.8–2.1)	24.17	26.92	1.7 (1.6–1.7)	1.3 (1.2–1.3)	24.09	25.34	1.5 (1.4–1.5)	1.3 (1.2–1.3)	23.99	18.36	2.6 (2.4–2.8)	2.3 (2.1–2.5)
32+	45.37	17.00	7.8 (7.4–8.2)	3.9 (3.6–4.1)	49.38	30.15	3.1 (3.0–3.2)	2.0 (1.9–2.1)	43.11	30.15	2.3 (2.2–2.4)	1.7 (1.7–1.8)	29.41	16.89	3.5 (3.2–3.7)	2.9 (2.7–3.1)
Specialist visits																
At least one SV	72.34	52.37	2.4 (2.3–2.5)	1.5 (1.5–1.6)	63.16	57.51	1.3 (1.2–1.3)	1.0 (0.9–1.0)	43.01	40.24	1.1 (1.1–1.2)	1.0 (0.9–1.0)	25.05	17.44	1.6 (1.5–1.7)	1.4 (1.3–1.5)
Total SV by classes																
0	27.66	47.63	1	1	36.84	42.49	1	1	56.99	59.76	1	1	74.95	82.56	1	1
1	14.98	18.66	1.4 (1.3–1.5)	1.1 (1.1–1.2)	17.95	18.99	1.1 (1.1–1.1)	1.0 (0.9–1.0)	18.53	17.93	1.1 (1.0–1.1)	1.0 (0.9–1.0)	13.41	10.08	1.5 (1.4–1.6)	1.4 (1.3–1.5)
2	10.20	10.27	1.8 (1.7–1.9)	1.2 (1.1–1.3)	11.17	11.97	1.1 (1.0–1.1)	0.9 (0.8–0.9)	8.75	8.46	1.1 (1.1–1.1)	1.0 (0.9–1.0)	5.02	3.41	1.7 (1.5–1.9)	1.4 (1.3–1.6)
3+	47.15	23.44	3.5 (3.4–3.7)	2.0 (1.9–2.1)	34.04	26.55	1.5 (1.5–1.5)	1.0 (1.0–1.1)	15.73	13.85	1.2 (1.2–1.3)	0.9 (0.9–1.0)	6.62	3.95	1.9 (1.7–2.1)	1.5 (1.4–1.7)
Laboratory tests																
At least one LT	85.31	66.10	3.0 (2.9–3.1)	1.8 (1.7–1.9)	84.35	69.28	2.4 (2.4–2.5)	1.8 (1.7–1.8)	76.95	57.36	2.5 (2.5–2.6)	2.1 (2.1–2.2)	66.17	37.03	3.4 (3.2–3.6)	3.0 (2.6–3.2)
Total LT by classes																
0	14.69	33.90	1	1	15.65	30.72	1	1	23.05	42.64	1	1	33.83	62.97	1	1
1–2	13.50	24.15	1.3 (1.2–1.4)	1.0 (1.0–1.1)	16.72	23.16	1.4 (1.4–1.5)	1.2 (1.2–1.3)	22.79	23.41	1.8 (1.8–1.9)	1.7 (1.6–1.7)	28.14	19.14	2.8 (2.6–3.0)	2.6 (2.4–2.8)
3–4	13.74	19.43	1.7 (1.6–1.8)	1.2 (1.1–1.3)	16.24	19.40	1.7 (1.6–1.8)	1.4 (1.3–1.5)	17.98	15.66	2.2 (2.1–2.3)	1.9 (1.8–2.0)	16.59	9.81	3.2 (2.9–3.5)	2.9 (2.6–3.1)
5+	58.07	22.52	6.2 (5.9–6.5)	3.1 (2.9–3.3)	51.38	26.73	4.0 (3.8–4.1)	2.6 (2.5–2.7)	36.18	18.30	3.8 (3.7–4.0)	2.9 (2.8–3.0)	21.44	8.09	5.0 (4.6–5.5)	4.3 (3.9–4.7)

adj: adjustment for Charlson Comorbidity Index.

Compared to survivors, the amount of hospital admissions among cases was markedly higher in the youngest age group (OR = 12.8; 95% CI 12.1–13.4) than the oldest one (OR = 4.7; 95% CI 4.4–5.1). The utilization of emergency departments followed a similar pattern, that is more stable in the youngest age group (OR = 7.1; 95% CI 6.8–7.4), with a decrease in the 95+ group (OR = 4.3; 95% CI 4.1–4.6). When primary care services (drug prescriptions, specialist visits, laboratory tests) are considered, the risks of utilization among cases and controls did not show a clear age-related pattern, with higher odds in the youngest and in the oldest age groups. Comorbidity adjusted ORs have shown a greater reduction, especially among the younger age groups.

Table 4 describes HCU stratified by the major causes of death. People who died from neoplasms, compared to their matched controls, had the highest risks of utilization of healthcare services, out of all the major causes of death considered in the analyses. No major differences were observed among subjects who died from cardiovascular or respiratory diseases.

**Table 4:** Healthcare utilization during the last year of life by major causes of death; Odds Ratios (OR) and 95% Confidence Intervals (CI) from conditional logistic regression for cases (decedents) and matched controls (survivors).

	Cancer (n = 92438)				Cardiovascular diseases (n = 124682)				Respiratory diseases (n = 29812)			
	Cases	Controls			Cases	Controls			Cases	Controls		
	%	%	OR (95% CI)	adj OR (95% CI)	%	%	OR (95% CI)	adjOR (95% CI)	%	%	OR (95% CI)	adjOR (95% CI)
Hospitalization admission			** **	** **			** **	** **			** **	** **
At least one HA	79.79	18.15	17.5 (16.7–18.4)	5.9 (5.6–6.3)	53.84	19.48	4.9 (4.7–5.0)	3.1 (3.0–3.2)	59.35	19.48	5.9 (5.5–6.3)	4.7 (4.4–5.0)
Total HA by classes												
0	20.21	81.85	1	1	46.16	80.52	1	1	40.65	80.52	1	1
1	29.27	12.76	9.1 (8.6–9.6)	4.6 (4.3–4.9)	27.02	13.94	3.4 (3.3–3.5)	2.6 (2.6–2.7)	28.14	13.75	3.7 (3.7–4.3)	3.7 (3.4–4.0)
2	21.75	3.66	24.2 (22.5–26.1)	8.6 (7.9–9.3)	13.80	3.82	6.4 (6.0–6.7)	4.2 (3.9–4.4)	15.64	4.00	7.6 (6.8–8.4)	6.7 (5.9–7.5)
3+	28.78	1.73	65.4 (59.5–72.0)	17.0 (15.3–19.0)	13.02	1.71	13.4 (12.5–14.4)	7.3 (6.7–7.9)	15.56	1.74	17.0 (14.7–19.6)	14.3 (12.2–16.8)
Emergency department visits												
At least one ED	70.40	24.01	8.1 (7.8–8.4)	3.8 (3.7–4.0)	58.99	26.08	4.2 (4.1–4.3)	2.9 (2.8–2.9)	64.27	26.63	4.9 (4.7–5.2)	3.8 (3.5–4.0)
Total ED by classes												
0	29.60	75.99	1	1	41.01	73.92	1	1	35.73	73.37	1	1
1	27.74	16.06	4.7 (4.5–4.9)	2.8 (2.7–3.0)	26.01	16.79	2.9 (2.8–3.0)	2.3 (2.2–2.4)	26.57	17.13	3.2 (3.0–3.4)	2.8 (2.6–3.0)
2+	42.67	7.95	15.0 (14.2–15.8)	5.9 (5.6–6.3)	32.98	9.29	6.7 (6.5–7.0)	4.1 (3.9–4.2)	37.70	9.49	8.1 (7.5–8.7)	5.8 (5.3–6.2)
Drug prescriptions												
At least one DP	97.40	86.98	5.6 (5.3–6.0)	3.3 (3.0–3.5)	93.83	85.09	2.7 (2.6–2.7)	2.2 (2.1–2.3)	94.06	85.90	2.7 (2.5–2.9)	2.2 (2.1–2.5)
Total DP by classes												
0–4	8.00	24.99	1	1	14.13	25.43	1	1	14.27	23.97	1	1
5–15	17.45	25.07	2.2 (2.1–2.3)	1.7 (1.6–1.8)	17.42	23.55	1.4 (1.3–1.4)	1.3 (1.2–1.3)	16.90	21.86	1.3 (1.2–1.4)	1.2 (1.1–1.3)
16–31	26.75	24.57	3.5 (3.4–3.7)	2.3 (2.1–2.4)	23.49	24.74	1.8 (1.7–1.8)	1.5 (1.5–1.6)	21.13	24.88	1.5 (1.4–1.6)	1.3 (1.2–1.4)
32+	47.80	25.36	6.4 (6.1–6.7)	3.3 (3.1–3.5)	44.97	26.28	3.3 (3.2–3.4)	2.5 (2.4–2.5)	47.71	29.29	2.9 (2.7–3.1)	2.2 (2.1–2.4)
Specialist visits												
At least one SV	73.81	52.02	2.7 (2.6–2.8)	2.0 (1.9–2.1)	46.39	43.85	1.1 (1.1–1.2)	0.9 (0.9–1.0)	46.89	45.48	1.1 (1.0–1.1)	0.9 (0.9–1.0)
Total SV by classes												
0	26.19	47.98	1	1	53.61	56.15	1	1	53.11	54.52	1	1
1	16.17	18.59	1.7 (1.6–1.7)	1.4 (1.4–1.5)	17.88	17.30	1.1 (1.1–1.1)	1.0 (1.0–1.0)	17.91	17.58	1.1 (1.0–1.1)	1.0 (0.9–1.1)
2	10.98	10.66	2.0 (1.9–2.1)	1.6 (1.5–1.7)	9.09	8.97	1.1 (1.0–1.1)	0.9 (0.9–1.0)	9.45	9.90	1.0 (0.9–1.1)	0.9 (0.8–1.0)
3+	46.66	22.77	4.0–3.9–4.2)	2.7 (2.6–2.8)	19.42	17.58	1.2 (1.1–1.2)	0.9 (0.9–0.9)	19.54	17.99	1.1 (1.1–1.2)	0.9 (0.9–1.0)
Laboratory tests												
At least one LT	91.96	65.24	6.4 (6.1–6.7)	4.5 (4.2–4.7)	74.63	59.65	2.0 (2.0–2.1)	1.7 (1.7–1.7)	77.47	60.71	2.3 (2.2–2.4)	2.0 (1.9–2.1)
Total LT by classes												
0	8.04	34.76	1	1	25.37	40.35	1	1	22.53	39.29	1	1
1–2	12.66	22.99	2.5 (2.4–2.6)	2.1 (2.0–2.2)	21.86	23.55	1.5 (1.5–1.6)	1.4 (1.3–1.4)	23.14	22.89	1.8 (1.7–1.9)	1.7 (1.6–1.8)
3–4	14.38	18.43	3.7 (3.5–3.9)	3.0 (2.8–3.2)	17.12	16.45	1.7 (1.7–1.8)	1.5 (1.5–1.6)	18.09	16.91	2.0 (1.8–2.1)	1.8 (1.7–2.0)
5+	64.92	23.82	13.6 (12.9–14.4)	8.4 (7.9–9.0)	35.65	19.65	3.1 (3.0–3.2)	2.3 (2.2–2.4)	36.25	20.92	3.3 (3.1–3.5)	2.6 (2.4–2.8)

adj: adjustment for Charlson Comorbidity Index.

Cases that died from cancer, compared to their survivors, showed highest risks of HCU. This was especially evident when considering the non-adjusted risk of having at least one hospital based acute healthcare service (HA OR = 17.5; 95% CI 16.7–18.4; ED OR = 8.1; 95% CI 7.8–8.4). ORs decreased when the analyses were performed, adjusting for comorbidities (HA OR = 5.9 95% CI 5.6–6.3; ED OR = 3.8 95% CI 3.7–4.0).

## Discussion

This study compared patterns of healthcare utilization among decedents in their last year of life and survivors, during the same period, in a large cohort of participants aged 55 years and older in the Friuli Venezia Giulia Region, Italy. The objective was to assess how age, proximity to death, sex and the major causes of death, influence the utilization of acute and primary care services. The results found, suggest that closeness to death is strongly associated to HCU in adults/elderly, but its influence varies between age groups and major causes of death. Since relatively few papers have studied healthcare utilization among decedents and survivors, we considered results also from studies that focused on healthcare costs. We assume that, considering each healthcare service separately, expenditure may be considered as a proxy of healthcare utilization even if there are, especially among acute healthcare services, important differences in costs [34].

Acute healthcare services play a large role in patient care at the end of life: 62% of dying patients receive hospital care and 63% access the ED at least once during the EOL. Decedents have higher risks of being hospitalized, compared to age-sex matched controls (HA OR = 7, ED OR = 5.2) and tend to be hospitalized longer (mean/median length of stay of: cases = 16.3/8 days, controls = 2.3/0 days).

In addition, the percentage of hospitalized subjects, in the last year of life, declines with the increase of age at death. A greater portion of younger-old decedents was admitted to the hospital, compared to the oldest-old. Furthermore, decedents aged 95 and above showed a marked reduction in the frequency of ED visits as oppose to other decedents. These results are consistent with those of other studies. Different studies, also showed how older age was associated with a lower frequency of acute hospital-based healthcare assistance, in the period preceding death [17,18,30]. This could be related to the fact that subjects in the oldest age group are often living in an institution and are therefore less likely to require hospital-based services. Another possible explanation may be that aggressiveness of medical care decreases with the increase of age. Some evidence suggested an increase in the use of healthcare in younger individuals already one year before the date of death, while in older individuals, the increase would occur only 4 months before death [9]. Therefore, by analyzing a one-year period before the index date, younger decedents could have a longer period of increased risk of hospital-based healthcare service utilization, with greater chances of being admitted to the hospital, more than once.

The high use of hospital-based services (HA and ED) at the EOL could be an indicator of a low quality of life in proximity to death. The worsening of clinical conditions that often precedes death, can indeed lead to an increase need of hospital-based healthcare assistance. Anyway, assessing the need of these services in the EOL, goes beyond the objectives of this study. Nevertheless, Gill et al suggested, by means of an observational study on individuals aged 70 years or above, during their last year of life, that acute illnesses and injuries leading to hospital admission play an important role in a disabling process, that leads to recurrent hospitalizations in more than half the decedents. For those patients, to enhance restorative interventions in the subacute, palliative care approach, home-care and outpatient setting should be considered [35]. Several studies showed that hospitalizations at the EOL can be potentially preventable [14,36]. Since EOL expenditures are driven largely by an increase in inpatient hospital costs [34], efforts should focus on reducing hospitalizations [37]. Hospital-at-home or in day-care services might be an alternative solution to hospitalizations, in certain situations [38]. This is also true, when considering elder patients,—not necessarily at the EOL—that are characterized by high frailty, which is responsible of the delivery of an important amount of acute healthcare services, which might often not be appropriate for this specific subgroup [39]. In Italy, a study conducted in the Regions of Emilia Romagna and Veneto seems to suggest that well-integrated palliative care approach can be effective in further reducing the percentage of patients who spend many days in hospital and/or undergo frequent and inappropriate changes of their care setting during the last month of life [40]. Those results were confirmed by other studies in patients with cancer, conducted in the same geographical areas [25,41]. These results underline how enhancing palliative care and reducing hospitalization during the EOL period were associated with an increase in patient and family satisfaction [41]. In Israel, Bentur et al had observed how the costs associated with the last 6 months of life in patients with metastatic cancer were lower for those who received acute care in addition to regular community care [13]. Without an extensive support network, there may be an increase in hospital-based services, used as “service substitutions” [16]. Since 55% of the individuals included in this study have died in hospital structures, all acute hospital-based services that were associated with the death of the patient, were excluded from analyses, to avoid imbalances due to the high frequency of this final contact.

Primary care as well, is an important determinant of healthcare assistance in the last year before death, with 87% of cases receiving more than five drug prescriptions, 54% requiring at least one specialist visit and 80% having at least one laboratory test. Controls, in contrast, had respectively: 75%, 47% and 62%. It has been estimated that these three services combined, represent almost 30% of healthcare expenditure in Italy [11]. These healthcare services also showed a progressively increasing trend of use in the EOL, although here age seems to play a more marginal role. Closeness to death is in fact associated with an increased use of primary care services. This pattern remains relatively constant across all ages, except for a slight U-shaped trend in drug prescriptions. Consistently with this finding, Moore et al, showed how proximity to death, rather than age itself, drives prescription expenditure in the population aged 70 or more [15]. Among decedents, the use of specialist visits and laboratory tests in the EOL showed a marked reduction with the increase of age. Pot et al showed a similar decrease of specialist visits with the increase of age among decedents aged 55 or above [19]. A possible cause, might be attributable to frailty markers such as poor cognitive ability [42], low educational level and low social support among the extremely elderly subjects, that may hamper access to appropriate care [19].

On the other hand, long-term-care use, which has not been analyzed in this work, has been found to increase in the last years of life [29]. Further studies are required to evaluate the different patterns in long-term care utilization, as oppose to primary care.

Another finding was that the risk of healthcare utilization among female decedents, compared to their matched controls were always lower than males’, especially for drug prescriptions, with the exception of laboratory tests. Other studies have shown lower hospital costs respectively for elderly [43] and decedent women, compared to men [44]. Further studies are needed to investigate whether healthcare assistance and needs in elderly woman are related to gender disparity or to true differences in healthcare needs, in the EOL, between men and women.

Specific causes of death, showed strong differences in HCU patterns, concerning both acute and primary care services. These were in fact consistently higher for patients dying from cancer, compared to those that died from cardiovascular and respiratory diseases. These findings are consistent with those from previous international studies on HCU in the EOL for cancer deaths, compared to all other causes of death [16]. A possible explanation may be that some of these diseases have a sudden clinical presentation (e.g. stroke, myocardial infarction and pneumonia), leading to a less intense healthcare service consumption. On the other hand, cancer has a longer course and requires more accesses to healthcare services. However, this does not explain the reduced likelihood of hospitalization for people who died due to chronic diseases (e.g. chronic obstructive pulmonary disease, heart failure). Another study noted a pattern of decreasing medical expenditure, frequency of hospital admission and utilization of intensive care units, with the increase of age, for all cause of death except cancer [45].

Considering the advanced age of the subjects that were analyzed, comorbidities are an important and frequent determinant of health, but they also play a relevant role in modifying life expectancy among younger individuals [46]. When adjusting the results from our analyses, according to the Charlson Comorbidity Index, the Odds Ratios decreased, with a sensible reduction among the younger decedents and the cancer patients. This is explainable, considering that comorbidities have a key role in defining the amount of healthcare services that an individual requires and have a great influence on survival.

Italy has the largest proportion of elderly in Europe, with more than one fifth of its residents, aged 65 years or above, at the beginning of 2015 (Eurostat http://ec.europa.eu/eurostat/data/database). This portion is expected to increase as the so-called “baby boom” cohort (those born between 1945 and 1964) ages progressively (Italian National Institute of Statistics [ISTAT], 2011). Despite this particular demographic conformation, there is limited knowledge about healthcare utilization in older age groups, in relation to the use at the EOL, in this country [11,39]. Atella and colleagues have analyzed for first in Italy primary healthcare expenditure, showing a faster increase in the healthcare costs as individuals approach the six-month period before death [11].

### Strengths and limitations of this study

To the best of our knowledge, this is the only article that compares the utilization among decedents and survivors, of acute hospital-based services, drug prescriptions, specialist’s visits and laboratory tests, in different age groups. All of the three studies [9,19,31] that showed the greatest similarities with ours, are not completely comparable. McGrail and coauthors studied the costs between decedents and survivors and does not focus on utilization itself. Forma and Pot analyzed long-term care and therefore include different sources from the ones used in this study. Our analysis distinguished the extremely elderly (≥95) in age stratification, as only few studies before have done [18,47,48]. Another advantage of this work is the use of regional, population-based databases, since the healthcare administrative data used for the analyses, comes from the entire Region of Friuli Venezia Giulia. This study has included in the analyses of risk of healthcare utilization, specialist visits, that have been scarcely evaluated in previous works [11,31]. This source could be used more broadly in future studies.

A limit of this study was that the overlook on primary care was not complete, since data regarding general practitioners’ visits are not collected systematically in the Region. Information on long-term care facilities and connected services (home assistance, home nursing, palliative care) were not included in the analyses, although they could provide a better understanding of institutionalized EOL periods and how this modifies the utilization of healthcare services (especially acute ones) mainly among the eldest. This goes beyond the objective of this study, but it could be considered in future works. Due to these limitations, it was also not possible to incorporate information regarding the place of death. That said, a European report has shown that only about 2% of the population aged 65 years or above, in Italy, is residing in long-term care facilities. This is in contrast with most European countries, where this percentage is greater than 5% [49]. Another limit is that the Charlson Comorbidity Index was calculated only by means of hospital discharge records. This served as a proxy for comorbidity, due to a lack of individual information on chronic conditions. Considering the possible incompleteness of said adjustment, greater attention has been given, throughout the article, to the value of unadjusted odds ratios.

## Conclusions

In conclusion, proximity to death is strongly associated to healthcare utilization in subjects aged 55 or above, but its influence varies between age groups and kind of service considered. Our results confirm that there is high use of acute hospital-based services in the last year of life, more markedly present in the younger-old. Further studies are needed to better understand how acute hospital-based healthcare use is influenced by the access to other primary and long-term-care services. A more complete overview on comorbidities could also help comprehend some other aspects influencing this complex phenomenon. Reducing the access to acute healthcare services is particularly important in an ageing population to improve the quality of life in proximity to death, with a consequent cost containment.
